# Music and Aging: Philosophically Speaking

**DOI:** 10.1093/geroni/igaf122.707

**Published:** 2025-12-31

**Authors:** Victor Fung

**Affiliations:** University of South Florida, Tampa, Florida, United States

## Abstract

The presenter will explore questions about humans being part of nature through classic Daoism and about human relationships across the lifespan through classic Confucianism. Like all living beings, aging in human is inevitable and is a natural process. Classic Daoists see that all phenomena are guided by Dao, which should be void of egoistic desires. Actions should be taken to support the natural way of being and growth. Music has a natural bond with humans since the earliest recorded history. The exchange of energies, yin and yang, within the music, within the human, and between the two becomes critical in maintaining a balance. An imbalance in this exchange could cause discomfort, unhappiness, illness, hopelessness, or other physical or psychological adversities. Classic Confucians focus on relationships and interactions among humans across the entire lifespan. Especially noteworthy is the role orientation in relation to the expected behaviors. Based on this role orientation and characteristics of different life stages, everyone in all stages of life could help to promote deep harmony. Based on these philosophical analyses, findings suggest that (a) all generations need to be better educated about the aging process, (b) music continues to have a special place in support of a high quality of life and a harmonious society, and (c) everyone’s understanding of the music of all living generations could promote a balanced, harmonious household, community, and society.

